# *N*′-[4-(Dimethyl­amino)­benzyl­idene]furan-2-carbohydrazide

**DOI:** 10.1107/S1600536810037244

**Published:** 2010-09-30

**Authors:** Yu-Feng Li, Fan-Yong Meng

**Affiliations:** a Microscale Science Institute, Department of Chemistry and Chemical Engineering, Weifang University, Weifang 261061, People’s Republic of China; b Weifang Middle School, Weifang 261061, People’s Republic of China

## Abstract

The title compound, C_14_H_15_N_3_O_2_, was prepared by the reaction of 4-(dimethyl­amino)­benzaldehyde and furan-2-carbohydrazide. The dihedral angle between the benzene ring and the furan ring is 25.59 (19)°. In the crystal, mol­ecules are linked by inter­molecular N—H⋯O hydrogen bonds, forming chains along [010].

## Related literature

For the applications of this class of Schiff base compounds, see: Habermehl *et al.* (2006 ▶); Nataliya *et al.* (2007 ▶). For a related structure, see: Li & Jian (2010 ▶).
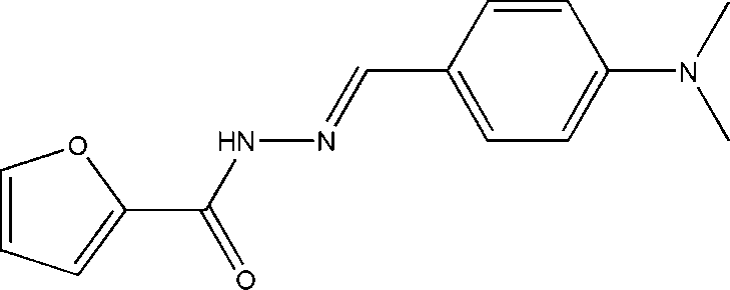


## Experimental

### 

#### Crystal data

C_14_H_15_N_3_O_2_
*M* *_r_* = 257.29
Orthorhombic, 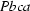

*a* = 10.866 (2) Å
*b* = 7.9654 (16) Å
*c* = 30.620 (6) Å
*V* = 2650.2 (9) Å^3^
*Z* = 8
Mo *K*α radiation
μ = 0.09 mm^−1^
*T* = 293 K
0.22 × 0.20 × 0.18 mm

#### Data collection

Bruker SMART CCD diffractometer
18964 measured reflections
2394 independent reflections
986 reflections with *I* > 2σ(*I*)
*R*_int_ = 0.172

#### Refinement

*R*[*F* ^2^ > 2σ(*F* ^2^)] = 0.051
*wR*(*F* ^2^) = 0.143
*S* = 0.75
2394 reflections
196 parameters
H atoms treated by a mixture of independent and constrained refinement
Δρ_max_ = 0.15 e Å^−3^
Δρ_min_ = −0.16 e Å^−3^

### 

Data collection: *SMART* (Bruker, 1997 ▶); cell refinement: *SAINT* (Bruker, 1997 ▶); data reduction: *SAINT*; program(s) used to solve structure: *SHELXS97* (Sheldrick, 2008 ▶); program(s) used to refine structure: *SHELXL97* (Sheldrick, 2008 ▶); molecular graphics: *SHELXTL* (Sheldrick, 2008 ▶); software used to prepare material for publication: *SHELXTL*.

## Supplementary Material

Crystal structure: contains datablocks global, I. DOI: 10.1107/S1600536810037244/lh5131sup1.cif

Structure factors: contains datablocks I. DOI: 10.1107/S1600536810037244/lh5131Isup2.hkl

Additional supplementary materials:  crystallographic information; 3D view; checkCIF report

## Figures and Tables

**Table 1:** Hydrogen-bond geometry (Å, °)

*D*—H⋯*A*	*D*—H	H⋯*A*	*D*⋯*A*	*D*—H⋯*A*
N2—H2*A*⋯O2^i^	0.86	2.10	2.933 (3)	163

Symmetry code: (i) 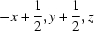.

**Fig. 1.**
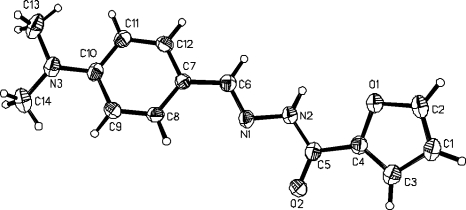
The molecular structure of the title compound showing 30% probability displacement ellipsoids and the atom-numbering scheme.

## supplementary crystallographic information

### Comment

Schiff bases bearing additional donor groups represent an important class of heteropolydentate ligands capable of forming mono-, bi-, and polynuclear complexes with metals in coordination chemistry (Nataliya *et al.*, 2007). They are important intermediates which have many interesting properties (Habermehl *et al.*, 2006). As part of our search for new schiff base compounds we synthesized the title compound (I), and the crystal structure is presented herein. The molecular structure of (I) is shown in Fig. 1. The dihedral angle between the benzene ring and the furan ring is 25.59 (19)°. In the crystal structure molecules are linked by intermolecular N-H···O hydrogen bonds to form one-dimensional chains along [010]. The bond lengths and angles agree with those observed in a related structure (Li & Jian, 2010).

### Experimental

A mixture of 4-(dimethylamino)benzaldehyde (0.1 mol), and furan-2-carbohydrazide (0.1 mol) was stirred in refluxing ethanol (20 mL) for 2 h to afford the title compound (0.089 mol, yield 89%). Single crystals suitable for X-ray measurements were obtained by recrystallization of solution of the title compound in ethanol at room temperature.

### Refinement

H atoms were fixed geometrically and allowed to ride on their attached atoms, with C—H = 0.97 Å, N-H = 0.86Å and *U*_iso_=1.2*U*_eq_(C,N). The H atoms of the methyl groups were refined independently with isotropic displacement parameters.

### Crystal data

**Table:**

C_14_H_15_N_3_O_2_	*F*(000) = 1088
*M**_r_* = 257.29	*D*_x_ = 1.290 Mg m^−^^3^
Orthorhombic, *P**b**c**a*	Mo *K*α radiation, λ = 0.71073 Å
Hall symbol: -P 2ac 2ab	Cell parameters from 986 reflections
*a* = 10.866 (2) Å	θ = 3.2–25.3°
*b* = 7.9654 (16) Å	µ = 0.09 mm^−^^1^
*c* = 30.620 (6) Å	*T* = 293 K
*V* = 2650.2 (9) Å^3^	Block, colorless
*Z* = 8	0.22 × 0.20 × 0.18 mm

### Data collection

**Table:**

Bruker SMART CCD diffractometer	986 reflections with *I* > 2σ(*I*)
Radiation source: fine-focus sealed tube	*R*_int_ = 0.172
graphite	θ_max_ = 25.3°, θ_min_ = 3.2°
φ and ω scans	*h* = −13→13
18964 measured reflections	*k* = −8→9
2394 independent reflections	*l* = −36→36

### Refinement

**Table:**

Refinement on *F*^2^	Primary atom site location: structure-invariant direct methods
Least-squares matrix: full	Secondary atom site location: difference Fourier map
*R*[*F*^2^ > 2σ(*F*^2^)] = 0.051	Hydrogen site location: inferred from neighbouring sites
*wR*(*F*^2^) = 0.143	H atoms treated by a mixture of independent and constrained refinement
*S* = 0.75	*w* = 1/[σ^2^(*F*_o_^2^) + (0.0687*P*)^2^] where *P* = (*F*_o_^2^ + 2*F*_c_^2^)/3
2394 reflections	(Δ/σ)_max_ = 0.002
196 parameters	Δρ_max_ = 0.14 e Å^−^^3^
0 restraints	Δρ_min_ = −0.16 e Å^−^^3^

### Special details

**Table:**

Geometry. All e.s.d.'s (except the e.s.d. in the dihedral angle between two l.s. planes) are estimated using the full covariance matrix. The cell e.s.d.'s are taken into account individually in the estimation of e.s.d.'s in distances, angles and torsion angles; correlations between e.s.d.'s in cell parameters are only used when they are defined by crystal symmetry. An approximate (isotropic) treatment of cell e.s.d.'s is used for estimating e.s.d.'s involving l.s. planes.
Refinement. Refinement of *F*^2^ against ALL reflections. The weighted *R*-factor *wR* and goodness of fit *S* are based on *F*^2^, conventional *R*-factors *R* are based on *F*, with *F* set to zero for negative *F*^2^. The threshold expression of *F*^2^ > σ(*F*^2^) is used only for calculating *R*-factors(gt) *etc*. and is not relevant to the choice of reflections for refinement. *R*-factors based on *F*^2^ are statistically about twice as large as those based on *F*, and *R*- factors based on ALL data will be even larger.

### Fractional atomic coordinates and isotropic or equivalent isotropic displacement parameters (Å^2^)

**Table:**

	*x*	*y*	*z*	*U*_iso_*/*U*_eq_	
N2	0.2524 (2)	0.2706 (3)	0.07906 (7)	0.0526 (7)	
H2A	0.2085	0.3518	0.0691	0.063*	
N1	0.2357 (2)	0.2125 (3)	0.12126 (7)	0.0522 (7)	
C9	0.2087 (3)	0.1579 (4)	0.25849 (9)	0.0565 (9)	
H9A	0.2642	0.0921	0.2740	0.068*	
O2	0.39675 (19)	0.0722 (3)	0.06431 (6)	0.0592 (6)	
C7	0.1388 (2)	0.2646 (4)	0.18944 (8)	0.0440 (7)	
C10	0.1164 (3)	0.2436 (4)	0.28088 (9)	0.0513 (8)	
O1	0.3004 (2)	0.4211 (3)	0.00187 (6)	0.0778 (8)	
C12	0.0460 (3)	0.3471 (4)	0.21190 (9)	0.0527 (8)	
H12A	−0.0101	0.4112	0.1962	0.063*	
C11	0.0337 (3)	0.3379 (4)	0.25639 (9)	0.0544 (9)	
H11A	−0.0300	0.3949	0.2702	0.065*	
C5	0.3391 (3)	0.1974 (4)	0.05371 (9)	0.0496 (8)	
N3	0.1068 (3)	0.2355 (4)	0.32592 (8)	0.0705 (9)	
C6	0.1552 (3)	0.2950 (4)	0.14317 (9)	0.0487 (8)	
H6A	0.1070	0.3751	0.1292	0.058*	
C14	0.1968 (7)	0.1475 (9)	0.35123 (15)	0.0956 (16)	
C8	0.2191 (3)	0.1689 (4)	0.21414 (9)	0.0517 (8)	
H8A	0.2819	0.1105	0.2002	0.062*	
C2	0.3436 (4)	0.4699 (6)	−0.03795 (11)	0.0939 (14)	
H2B	0.3175	0.5649	−0.0529	0.113*	
C4	0.3625 (3)	0.2785 (4)	0.01224 (9)	0.0533 (9)	
C1	0.4275 (4)	0.3640 (5)	−0.05229 (11)	0.0846 (13)	
H1B	0.4701	0.3704	−0.0786	0.101*	
C13	0.0192 (5)	0.3396 (9)	0.34859 (14)	0.0881 (15)	
C3	0.4401 (3)	0.2390 (4)	−0.01997 (10)	0.0698 (10)	
H3A	0.4925	0.1467	−0.0209	0.084*	
H13A	0.025 (4)	0.306 (5)	0.3795 (14)	0.127 (16)*	
H13B	−0.057 (5)	0.330 (7)	0.3362 (15)	0.15 (2)*	
H14A	0.202 (4)	0.030 (7)	0.3405 (13)	0.13 (2)*	
H13C	0.038 (6)	0.456 (8)	0.3456 (16)	0.18 (3)*	
H14B	0.168 (4)	0.141 (6)	0.3805 (17)	0.148 (19)*	
H14C	0.273 (7)	0.198 (11)	0.352 (2)	0.24 (4)*	

### Atomic displacement parameters (Å^2^)

**Table:**

	*U*^11^	*U*^22^	*U*^33^	*U*^12^	*U*^13^	*U*^23^
N2	0.0640 (17)	0.0533 (18)	0.0406 (13)	0.0055 (14)	0.0054 (12)	0.0084 (12)
N1	0.0624 (17)	0.0508 (18)	0.0435 (14)	−0.0018 (14)	0.0053 (12)	0.0071 (12)
C9	0.069 (2)	0.048 (2)	0.0520 (18)	0.0090 (17)	0.0001 (16)	0.0086 (15)
O2	0.0723 (15)	0.0515 (16)	0.0537 (13)	0.0078 (12)	0.0064 (11)	0.0064 (11)
C7	0.0477 (18)	0.041 (2)	0.0434 (16)	0.0008 (15)	0.0062 (13)	0.0015 (14)
C10	0.0603 (19)	0.049 (2)	0.0442 (16)	−0.0075 (16)	0.0075 (15)	−0.0022 (16)
O1	0.105 (2)	0.074 (2)	0.0543 (14)	0.0284 (16)	0.0211 (13)	0.0159 (12)
C12	0.055 (2)	0.049 (2)	0.0538 (19)	0.0067 (16)	−0.0031 (15)	0.0056 (15)
C11	0.050 (2)	0.062 (2)	0.0518 (18)	0.0065 (17)	0.0087 (15)	−0.0008 (15)
C5	0.060 (2)	0.046 (2)	0.0423 (17)	−0.0045 (18)	0.0037 (15)	−0.0013 (14)
N3	0.090 (2)	0.077 (2)	0.0447 (15)	0.0085 (18)	0.0055 (15)	−0.0008 (15)
C6	0.0541 (19)	0.042 (2)	0.0501 (18)	−0.0054 (16)	0.0021 (15)	0.0044 (14)
C14	0.130 (5)	0.101 (5)	0.055 (3)	0.018 (4)	−0.016 (3)	0.010 (3)
C8	0.060 (2)	0.042 (2)	0.0536 (19)	0.0105 (16)	0.0087 (15)	0.0031 (14)
C2	0.130 (4)	0.093 (4)	0.058 (2)	0.029 (3)	0.029 (2)	0.034 (2)
C4	0.068 (2)	0.046 (2)	0.0462 (18)	0.0010 (17)	0.0017 (15)	0.0016 (15)
C1	0.116 (3)	0.086 (3)	0.052 (2)	0.021 (3)	0.024 (2)	0.013 (2)
C13	0.092 (4)	0.122 (5)	0.051 (2)	0.000 (3)	0.016 (2)	−0.018 (2)
C3	0.086 (3)	0.066 (3)	0.058 (2)	0.012 (2)	0.0147 (18)	0.0049 (18)

### Geometric parameters (Å, °)

**Table:**

N2—C5	1.352 (4)	C5—C4	1.447 (4)
N2—N1	1.384 (3)	N3—C14	1.431 (5)
N2—H2A	0.8600	N3—C13	1.441 (5)
N1—C6	1.283 (3)	C6—H6A	0.9300
C9—C8	1.366 (4)	C14—H14A	0.99 (5)
C9—C10	1.393 (4)	C14—H14B	0.95 (5)
C9—H9A	0.9300	C14—H14C	0.92 (7)
O2—C5	1.222 (3)	C8—H8A	0.9300
C7—C8	1.384 (4)	C2—C1	1.317 (5)
C7—C12	1.385 (4)	C2—H2B	0.9300
C7—C6	1.448 (4)	C4—C3	1.336 (4)
C10—C11	1.391 (4)	C1—C3	1.410 (5)
C10—N3	1.385 (3)	C1—H1B	0.9300
O1—C4	1.359 (4)	C13—H13A	0.99 (4)
O1—C2	1.363 (4)	C13—H13B	0.92 (5)
C12—C11	1.371 (4)	C13—H13C	0.95 (6)
C12—H12A	0.9300	C3—H3A	0.9300
C11—H11A	0.9300		
			
C5—N2—N1	118.9 (3)	C7—C6—H6A	119.7
C5—N2—H2A	120.6	N3—C14—H14A	109 (3)
N1—N2—H2A	120.6	N3—C14—H14B	108 (3)
C6—N1—N2	114.0 (3)	H14A—C14—H14B	106 (4)
C8—C9—C10	121.2 (3)	N3—C14—H14C	115 (5)
C8—C9—H9A	119.4	H14A—C14—H14C	112 (6)
C10—C9—H9A	119.4	H14B—C14—H14C	107 (4)
C8—C7—C12	116.7 (3)	C9—C8—C7	121.7 (3)
C8—C7—C6	123.3 (3)	C9—C8—H8A	119.1
C12—C7—C6	119.8 (3)	C7—C8—H8A	119.1
C11—C10—C9	117.7 (3)	C1—C2—O1	110.7 (3)
C11—C10—N3	120.9 (3)	C1—C2—H2B	124.6
C9—C10—N3	121.4 (3)	O1—C2—H2B	124.6
C4—O1—C2	106.0 (3)	C3—C4—O1	109.7 (3)
C11—C12—C7	122.6 (3)	C3—C4—C5	130.8 (3)
C11—C12—H12A	118.7	O1—C4—C5	119.4 (3)
C7—C12—H12A	118.7	C2—C1—C3	106.6 (3)
C12—C11—C10	120.1 (3)	C2—C1—H1B	126.7
C12—C11—H11A	119.9	C3—C1—H1B	126.7
C10—C11—H11A	119.9	N3—C13—H13A	105 (3)
O2—C5—N2	123.8 (3)	N3—C13—H13B	111 (3)
O2—C5—C4	120.5 (3)	H13A—C13—H13B	116 (4)
N2—C5—C4	115.7 (3)	N3—C13—H13C	112 (4)
C10—N3—C14	120.7 (3)	H13A—C13—H13C	110 (4)
C10—N3—C13	120.2 (3)	H13B—C13—H13C	103 (5)
C14—N3—C13	118.2 (4)	C4—C3—C1	106.9 (3)
N1—C6—C7	120.7 (3)	C4—C3—H3A	126.6
N1—C6—H6A	119.7	C1—C3—H3A	126.6

### Hydrogen-bond geometry (Å, °)

**Table:**

*D*—H···*A*	*D*—H	H···*A*	*D*···*A*	*D*—H···*A*
N2—H2A···O2^i^	0.86	2.10	2.933 (3)	163

Symmetry codes: (i) −*x*+1/2, *y*+1/2, *z*.
